# An optimal filter for short photoplethysmogram signals

**DOI:** 10.1038/sdata.2018.76

**Published:** 2018-05-01

**Authors:** Yongbo Liang, Mohamed Elgendi, Zhencheng Chen, Rabab Ward

**Affiliations:** 1 School of Electrical and Computer Engineering, University of British Columbia, Vancouver, V6T 1Z4, Canada; 2 School of Electronic Engineering and Automation, Guilin University of Electronic Technology, Guilin 541004, PR China; 3 School of Life and Environmental Sciences, Guilin University of Electronic Technology, Guilin 541004, PR China; 4 Faculty of Medicine, University of British Columbia, Vancouver, V6T 1Z3, Canada; 5 BC Children's & Women's Hospital, Vancouver, V6H 3N1, Canada

**Keywords:** Biomarkers, Medical research, Cardiovascular diseases

## Abstract

A photoplethysmogram (PPG) contains a wealth of cardiovascular system information, and with the development of wearable technology, it has become the basic technique for evaluating cardiovascular health and detecting diseases. However, due to the varying environments in which wearable devices are used and, consequently, their varying susceptibility to noise interference, effective processing of PPG signals is challenging. Thus, the aim of this study was to determine the optimal filter and filter order to be used for PPG signal processing to make the systolic and diastolic waves more salient in the filtered PPG signal using the skewness quality index. Nine types of filters with 10 different orders were used to filter 219 (2.1s) short PPG signals. The signals were divided into three categories by PPG experts according to their noise levels: excellent, acceptable, or unfit. Results show that the Chebyshev II filter can improve the PPG signal quality more effectively than other types of filters and that the optimal order for the Chebyshev II filter is the 4^th^ order.

## Introduction

With the last few years advancements in communication and information technology, wearable health devices have emerged in recent years and are designed to obtain physiological information and measurements from the human body^1^. Despite these developments, little progress has been made toward fast, stable, and accurate signal-filtering techniques for wearable devices^2–4^.

The photoplethysmogram (PPG) is an important physiological signal of the human cardiovascular system and serves as the external manifestation of cardiac contractions within the dense arteriovenous network^5,6^. The PPG signal is extracted via an LED transmitter, which generates a red or infrared light that illuminates the skin on the fingertip, earlobe, or forehead. A photosensitive diode is used to measure the light absorbed by the body’s tissue over time, and these measurements can reflect changes in blood volume. The common PPG acquisition methods include transmission and reflection^7,8^; therefore, the PPG represents the aggregated expression of many physiological processes within the cardiovascular system^9^. The PPG is a high-fusion signal that measures activity during the heart’s systolic and diastolic periods, hemodynamics^10,11^, hemorheology^12^, and the peripheral microcirculation system^5,13,14^. PPGs can be easily extracted from human peripheral tissue^15^, such as fingers, toes, earlobes, wrists, and the forehead; therefore, they have great potential for application in wearable health devices.

Many physiological parameters have been studied and extracted using the PPG technique, such as heart rate, blood–oxygen saturation, respiration rate, blood pressure, and arterial stiffness; however, its practical applications are limited in wearable health equipment. There is a need for research that evaluates and compares the impact of different filter types on PPG signal quality. Such investigations could have a significant impact on the development of wearable health devices, consequently improving health monitoring, diagnosis, and screening techniques.

A comparative study^16^ of the PPG signal quality index (SQI) was conducted and found that the skewness method (S_SQI_), which can be used to distinguish between excellent, acceptable, and unfit signals, is optimal for evaluating PPG signal quality. The S_SQI_ method is also able to make the systolic and diastolic waves more salient in the filtered PPG signal, which allows for real-time and low-computation signal processing in wearable health devices. Note, simpler SQIs (such as the S_SQI_ method) that achieve the same or even higher accuracy than complex SQIs are necessary for global health applications^17^ and for speeding up the data analysis and transmission^18^.

Based on the findings in (ref. 16), the S_SQI_ can be used to assess and evaluate PPG-filtering methods in order to identify the optimal filter type for short recorded PPGs. Identifying the optimal filter type would be useful for wearable health devices because the PPG is a weak, low-frequency physiological signal that is more susceptible to noise interference^19^. Therefore, this paper investigates and compares different PPG signal filter methods by analyzing the performances of each filter type and its order. The S_SQI_ method is used as the evaluating criterion.

## Results

**Figure 1:**
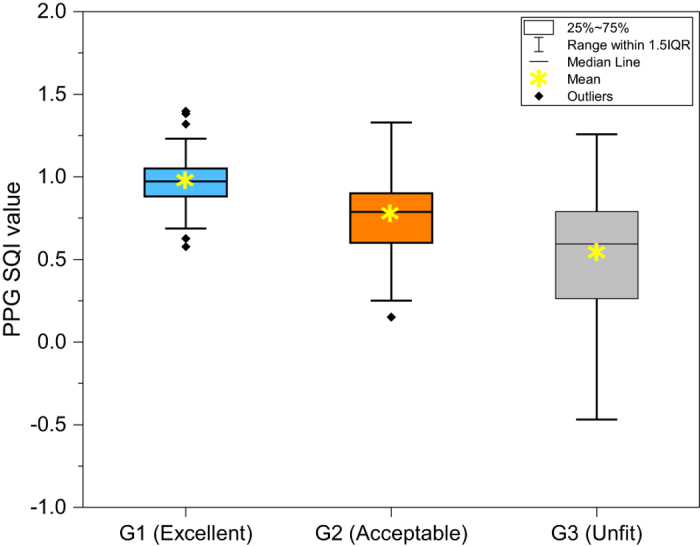
A box plot of the signal quality index of raw photoplethysmogram signals.

To analyze the filtering performance of the PPG signal, three categories of signal quality were adopted. G1 (excellent; 36 subjects) contains the complete contraction and diastolic period with the dicrotic wave and tidal wave; G2 (acceptable; 132 subjects) contains the complete contraction period, but the dicrotic wave and tidal wave were polluted by noise, and G3 (unfit; 51 subjects) only contains noise. For each group, the S_SQI_ of the raw PPG signals were calculated. Figure 1 illustrates a box plot of the raw PPG S_SQI_ for G1, G2, and G3, which indicates different levels of signal quality between each group.

**Figure 2:**
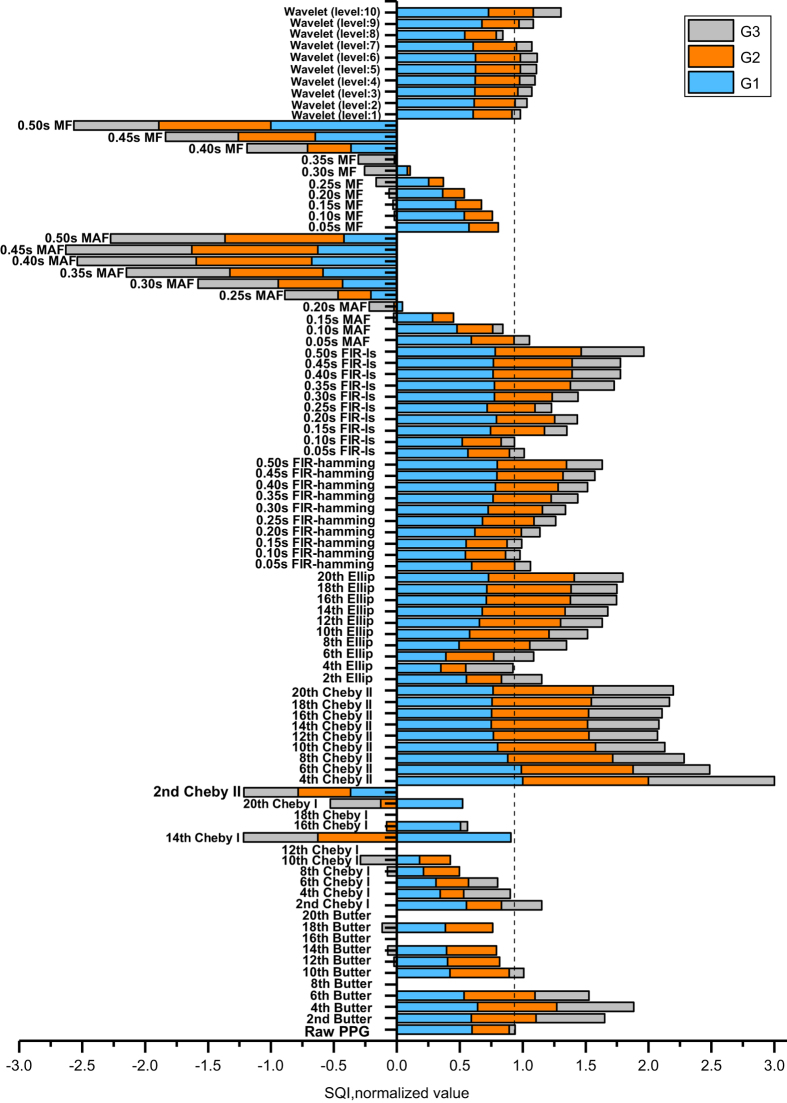
A stacked histogram of normalized signal quality index (SQI) values for all filter types and orders. The SQI was calculated using skewness.

**Figure 3:**
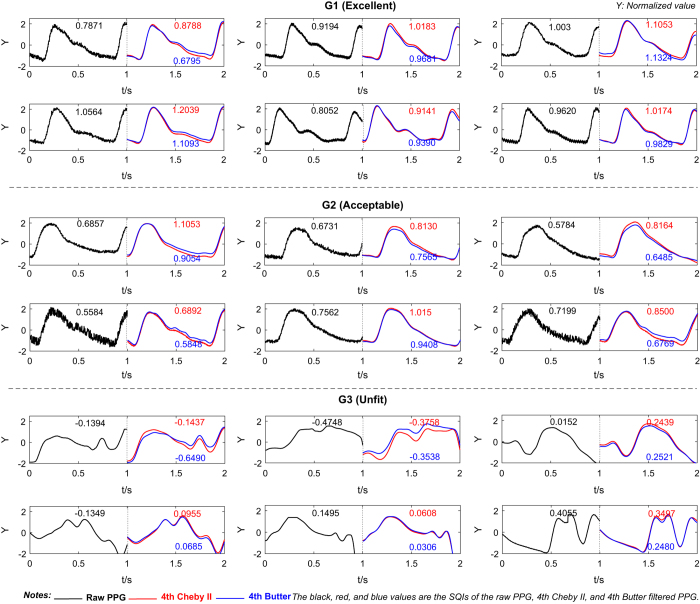
A comparison of raw and filtered photoplethysmogram (PPG) waveforms in G1, G2, and G3 classes. The skewness signal quality index values are shown for the raw PPG waveform (black color), the 4^th^-order Cheby II (red color), and 4^th^-order Butterworth (blue color) filters.

The S_SQI_ of the filtered PPG signals were calculated. To compare the filter types, an average S_SQI_ was calculated for each of the five orders of each filter type. The S_SQI_ method has shown a good distinction performance for these three groups of PPG waveforms, confirming the finding in (ref. 16). More than half of the filters were found to improve the signal quality. Figure 2 displays a stacked histogram of the SQI for G1, G2, and G3, which details the different filters and orders. Compared to other orders of Cheby II and other filters, the 4^th^-order Cheby II was the most effective at improving the PPG SQI. Figure 3 shows a comparison between raw PPG waveforms and those processed through the 4^th^-order Cheby II and 4^th^-order Butterworth filters.

## Discussion

In recent years, BP estimation based on only PPG signal has been an increasingly interesting area of focus for many researchers^20–22^. The morphology features^23^ of high quality PPG signals can be extracted beat by beat to predict the continuous cuff-less blood pressure. In our study, many precautions were adopted during the data collection and data archiving period. Abundant physiological information was recorded, and strict data collection requirements were implemented to avoid interference and noise as much as possible. The purpose of this series study was to explore and estimate the BP prediction model based on high quality and pure PPG signals. Thus, three PPG segments for each participant. Based on the findings in (refs 16,24), we selected a 2-second length, and our recently published paper^25^ discusses the rationale of this length choice.

Research in the area of PPG signal analysis is increasing, particularly for use in health monitoring, screening, and diagnosis devices. However, due to the complexity of the PPG signal and the diversity of usage scenarios for wearable health devices, PPG signal processing is confronted by issues of strong interference and high noise. Researchers^16,26,27^ have carried out a number of studies in this area, verifying that morphological information obtained from PPGs change when different signal-acquisition processes are used. However, there are no comparative studies that examine what the optimal filtering method for PPG signals is. Most researchers in this field have focused on optimization and performance analysis of certain filtering methods for a variety of purposes. For example, some explored continuous and real-time signal processing, some focused on removing strong motion noise^28–30^, and some have developed different filter types^31–34^.

Filter performance is typically evaluated by analyzing the filter parameters and frequency response and observing the noise change before and after filtering to judge the advantages and disadvantages of the filter. This can be challenging in that conclusions on the performance of the filter may differ due to the experience and other subjective characteristics of the researcher, which may lead to misleading results that can affect the accurate analysis of real signals. Especially for the complex and highly fused PPG signal, determining an optimal filter is very important to the analysis of its physiological and pathological significance.

We know that PPG signals reveal the aggregated systolic and diastolic activities of the heart^35^, the state of the vascular system (e.g., health, aging, disease, etc.)^36,37^, the differences between the microcirculation systems of different populations, gender^38^, gravity (i.e., the vertical distance from the heart to the PPG sensor)^39^, muscle jitter, movement, white noise, and other measurements. In addition to physiological information, PPG signals also contain some interference and noise, which not only necessitates control of the signal-acquisition process but also proper, effective filtering. Different filtering methods are chosen according to their purpose.

To determine the optimal filter and order, the filtering performance of multiple filter types on the PPG signal must be compared. In the present study, nine types of filters with five different orders were used to filter 219 (2.1s) PPG signals, which were categorized as G1, G2, or G3. To compare and analyze changes in signal quality, the PPG signal was processed through the five orders of each filter type, and the mean S_SQI_ was calculated from the results. The S_SQI_ also reflected the different filter performances: when the processed PPG signals were compared to the raw signals, some filters were found to significantly improve signal quality, while others severely reduced signal quality. To analyze this change more clearly, the SQI values were normalized (see Fig. 2). From the histogram of normalized SQI values, it can be observed that the Chebyshev II filter greatly improved the signal quality of G1, G2, and G3. Although there are differences between the filtering methods used in Fig. 2, Chebyshev II remained the optimal filter for the PPG signal.

As previously mentioned, the Butterworth and Chebyshev I filters are the two most commonly used types. In addition, they require lower orders than other filters to achieve the same performance levels. However, these filters are not without their disadvantages. Chebyshev I is an equal-ripple bandpass filter, which may adversely affect its ability to filter signals carrying abnormality related morphology. The Chebyshev II and elliptic filters have sharper frequency transition zones compared to the Butterworth and Chebyshev I filters. However, similar to the Chebyshev I filter, elliptic filters have an equal ripple in both the passband and the stopband, which negatively impacts the morphology of the filtered PPG waveform morphology.

The Chebyshev II filter not only has a sharper transition zone but its passband is also flat and contains no ripples, although there is an equal ripple in the stopband. These characteristics ensure that the useful component of the signal in the passband is affected as little as possible. Therefore, because Chebyshev II has excellent frequency selectivity and no equal ripple in the passband, it can filter out interference and noise while maintaining the valuable information of the signal, as can be seen in Fig. 3.

For the signal quality data, we concluded that the Chebyshev II filter improves the PPG signal quality more effectively than other filters, and therefore we only made a comparison between the Butterworth filter (the gold standard filter) and the Chebyshev II filter (the optimal filter according to the results obtained from Fig. 2) as shown in Fig. 3. In other words, from the mean SQI perspective, the Cheby II and Butter showed better results as seen in Fig. 2. Cheby II is the optimal filter because of better frequency selectivity and flat passband. From the detailed cases shown in Fig. 3, Cheby II improved most of the SQIs in all categories (G1, G2 and G3). We can also see that the high-frequency and baseline drift were filtered.

It is interesting that the lower order filter was able to capture the main events of the PPG waveform. This finding is in agreement with previously published works^40,41^ showing that the lower the order, the better the filter performance in analyzing biomedical signals. Moreover, it is always preferable to have a filter with a lower in order to achieve less computation time (i.e. consuming less CPU power because of the shorter computation time), as reported in (ref. 42), especially if the digital filter is part of a battery driven wearable monitoring device. Although higher orders can sharpen the transition zones in the frequency domain, they can cause adverse effects (please refer to the results of high order filters in Fig. 2).

With the advent of wearable technology, many physiological signals can now be quickly extracted from the human body. However, due to the variety of wearable devices available today that collect, save, and transmit these signals, there is inconsistency in filtering methods, leading to contradictory filtering accuracy, to obtain high fidelity data. Consequently, researchers and companies are paying greater attention to the filtering and de-noising methods for signal processing in order to improve the signal quality. The comparison and evaluation of filtering performance has practical significance for the selection of filters.

In the present research, 90 filter configurations, comprised of 9 filter types with 10 different orders, were used in a comparative analysis that evaluated the performance of each PPG filter type. A total of 219 (2.1s) PPG short recordings were collected and subsequently divided by two PPG annotation experts into three categories of signal quality, according to their noise level: excellent (G1), acceptable (G2), and unfit (G3). The PPG SQI was used to evaluate filter performance. The optimal filter type and order were determined by analyzing the SQI data collected from the filtered PPG signals. This study found that the Chebyshev II filter can improve the PPG signal quality more effectively than other types of filters, and the optimal order for this filter is the 4^th^ order.

Obtaining high-quality, low-noise PPG signals is an essential step in the accurate events detectionand evaluation of physiological parameters, thereby improving health monitoring, screening, and diagnosis methods^43,44^. The proposed filter can be implemented into the design of portable/wearable health devices and smartphone applications and, consequently, can improve the early diagnosis and treatment of diseases.

## Methods

### Experimental design and data acquisition

Data was collected from 219 subjects (104 males and 115 females; age: 57±15 years; height: 161±8 cm; weight: 60±11 kg) recruited from Guilin People’s Hospital. Potential subjects who suffered from diseases other than cardiovascular diseases (CVDs), such as neurological disorders, were excluded. In the experiment, the subjects first waited in a rest area for more than 30 minutes before entering the data collection room. After entering the room, the subjects sat in the most comfortable posture on a chair with a backrest and placed their arms flat on an empty tabletop. The experiment operators used a customized probe (set to a sampling rate of 1 kHz and a 12-bit AD precision conversion) to collect the PPG signal of the left index finger, as well as the Omron 7201 electronic sphygmomanometer to measure the blood pressure in the right forearm. Both measurements were taken simultaneously and were completed within three minutes. Three records of 2.1-second 12-bit AD PPG waveform data were stored in an accompanying app for each subject. The basic physiological information for each subject, including gender, age, height, and weight, was also collected through the app.

Table 1 displays the subjects’ demographic data and physiological parameters. Most of the subjects were elderly, with a mean age of 57 years, and the proportion of those over 50 years was 74.4%. In addition, the dataset did not include only healthy subjects; 39.2% of the subjects had been diagnosed with CVDs (i.e., hypertension: 26%; vascular infarction: 13.2%). Note that as the subjects aged and experienced pathological changes in their organs or tissues, the information fused in the PPG signal became richer and more complex; therefore, valuable data can be extracted from successful noise filtering.

### Data annotation

Two independent researchers annotated the PPG signals (657 PPG recordings, 2.1 s each) based on three groups: Group 1 (G1) corresponded to “excellent” for diagnosis, Group 2 (G2) corresponded to “acceptable” for diagnosis, and Group 3 (G3) corresponded to “unfit” for diagnosis. The annotation process was carried out according to the recommendations in (ref. 16), and one annotation file for all PPG signals was generated after adjudicating the discrepancies between the annotators.

### Data analysis

Some studies consider the perfusion index (P_SQI_) to be the gold standard for assessing PPG signal quality. For simpler, more accurate evaluations of signal quality, various methods have been proposed. In ref. 16, eight signal quality indices were compared: P_SQI_, S_SQI_, kurtosis (K_SQI_), entropy (E_SQI_), signal-to-noise ratio (N_SQI_), zero-crossing (Z_SQI_), matching of multiple systolic wave detection algorithms (M_SQI_), and relative power (R_SQI_). For PPG waveforms with lengths ranging from 1s to 30s, the S_SQI_ method performs better than the others. When 2s is used as the window of the PPG waveform segment, the classification of excellent from unfit waveforms is best using this method. Krishnan *et al*^45^. introduced and tested skewness and found that the S_SQI_ was connected to the quality of the PPG waveform. Other researchers validated this observation, such as in (ref. 16), who found that S_SQI_ is the optimal method for assessing PPG signal quality. Skewness is used to measure the probability distribution of symmetrical waveforms, which is calculated as follows:

$$S_{\mathrm{SQI}}=1/N\sum_{i=1}^{N}{\left[x_{i}-{\hat{µ}}_{x}/\sigma \right]}^{3},$$

where *N* is the number of PPG signals, and ${\hat{µ}}_{x}$ and σ are the empirical estimates of the mean and standard deviation of *x*_*i*_, respectively.

**Figure 4:**
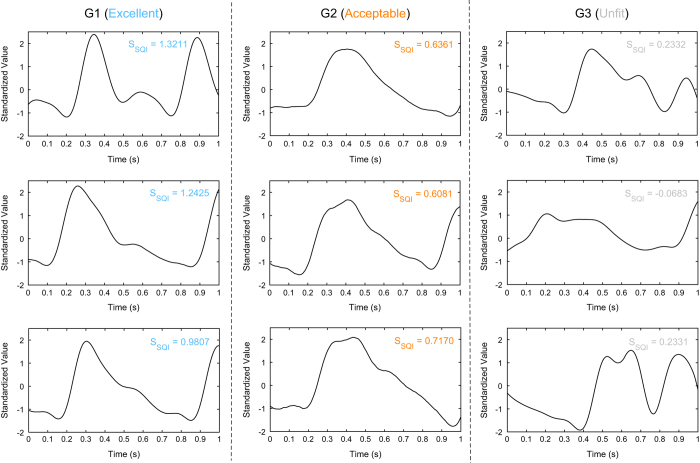
Photoplethysmogram waveform classifications. Note, the SQI here is calculated using the skewness.

**Figure 5:**
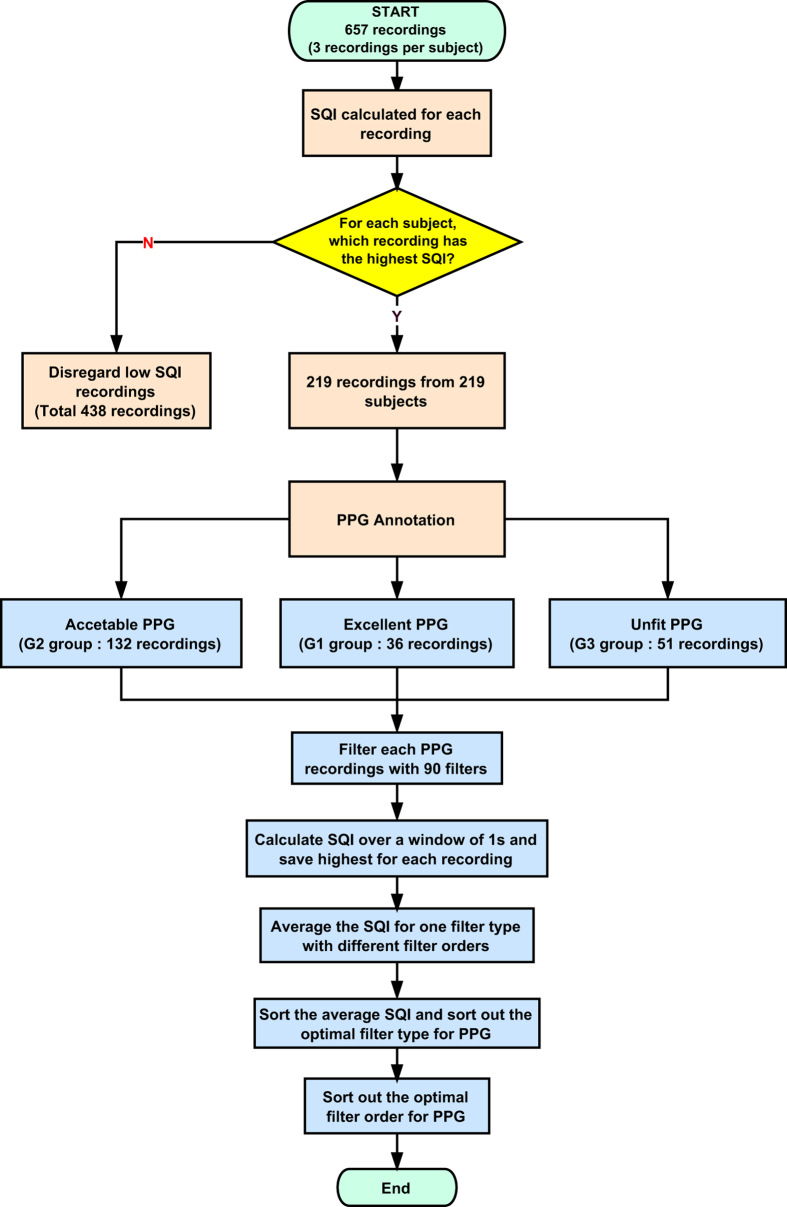
The photoplethysmogram study flowchart. PPG stands for photoplethysmogram; SQI stands for signal quality index.

This paper analyzes and compares the raw and filtered PPG signals to determine which configuration of filter type and order is most effective at reducing noise and improving signal quality. The S_SQI_ values were calculated for each 1s window of the recorded PPG waves, and the highest value for each record was determined to be the S_SQI_ resulting from the associated filter type and order. The PPG waves were then classified into one of three groups, according to the level of noise: excellent (G1), acceptable (G2), or unfit (G3). Excellent waves contained the complete heart contraction and diastolic period with typical PPG information, such as the tidal wave and the dicrotic wave. Acceptable waves contained the complete heart contraction period, but some PPG information was distorted and polluted by noise. Finally, unfit waves contained enough noise that it was impossible to distinguish the heartbeat period. Figure 4 shows a schematic of the SQI calculations and PPG wave classifications for one recording. Figure 5 shows the PPG signal recording flowchart used in this study.

Data collection was conducted according to the ethics rules and regulations of Guilin People Hospital and the Guilin University of Electronic Technology in China and was approved by the ethics committee. Informed consent was acquired from participants before the data collection process was initiated. The background of the entire project can be found in (ref. 25). The dataset has been fully uploaded to the network, and users can download via Data Citation 1.

### Filter Design

As far as we know, no study so far has systematically explored different orders for PPG filters. Therefore, we systematically investigated filter orders with the aim of improving the morphology of the PPG waveforms. After an initial literature review, multiple filter methods with different orders and designs were selected to compare filter performance. In each cited paper for each filter type, the detailed filter and order were reported. This information helped in the selection of comparative filter types and orders. Nine filter types in total were selected, each with ten filter orders. Each set of filter orders included ten valued that sequentially increased at a fixed rate (e.g., Moving average filter increased by 0.05; thus generating a filter order set {0.05, 0.10, 0.15,...,0.5}, etc). A total of 90 filter configurations were produced which helped to determine the optimal filter order.

Note that the filters designed in our filter study were all digital filters and not hardware filters. All filters were implemented using Matlab software version R2017a (The MathWorks, Inc., MA, USA). Moreover, PPG signal annotation, normalization and signal quality assessment were carried out using the same software. Filters were designed and configured based on the Signal Process Toolbox of MATLAB. A laptop is employed in this study which is configured as 2.7 GHz CPU and 8GB RAM. The following subsections describe each filter type and order.

### Moving-average filter (MAF)

An MAF filter with a window length of 3 samples was used to filter PPG signals in (ref. 46) without any justification; therefore, we investigated different window lengths of 0.05 s, 0.10 s, 0.15 s, 0.20 s, 0.25 s, 0.30 s, 0.35 s, 0.40 s, 0.45 s and 0.50 s. Note the units of length here are in seconds, not in data samples, to ensure scalability and repeatability as recommended in (ref. 47). The Matlab function used to implement this step was *smooth*.

### Median filter (MF)

An MF filter with a window length of 3 samples was used to filter PPG signals in (ref. 48) without justifying the chosen length. Therefore, we investigated lengths of 0.05 s, 0.10 s, 0.15 s, 0.20 s, 0.25 s, 0.30 s, 0.35 s, 0.40 s, 0.45 s and 0.50 s. The Matlab function used to implement this step was *medfilt1*.

### Finite impulse response filter (FIR-hamming and FIR-ls)

An FIR filter of order 10 was used to filter PPG signals in (ref. 49). No justification of the order was provided; therefore, we investigated lengths of 0.05 s, 0.10 s, 0.15 s, 0.20 s, 0.25 s, 0.30 s, 0.35 s, 0.40 s, 0.45 s and 0.50 s. Meanwhile, hamming window (FIR-hamming) method and least squares (FIR-ls) method are adopted in FIR filter design. The Matlab functions used to implement these two filters were *fir1* and *designfilt*, respectively.

### Butterworth filter (Butter)

A butter filter of order 2 was used to filter PPG signals in (refs 50–52). The explanation of the order choice was not provided, and as a result, we investigated orders set to 2, 4, 6, 8, 10, 12, 14, 16, 18, and 20. It is known, for IIR bandpass and bandstop filter design, an even order (2*N) is an appropriate choice as Matlab manual. The Matlab function used to implement this step was *butter*.

### Chebyshev filter (Cheby I and Cheby II)

A Cheby filter Type-I of order 6 was used to filter PPG signals in (ref. 53), and Type-II of order 5 was also used in (ref. 54). Because of the lack of justification of the order value, we investigated orders 2, 4, 6, 8, 10, 12, 14, 16, 18 and 20. Therefore, Type I and Type II Cheby filters are designed and the same orders as Butter filter are adopted to compare. The Matlab functions used to implement these two filters were *cheby1* and *cheby2*, respectively.

### Elliptic filter (Ellip)

An ellip filter of order 4 was used to filter PPG signals in (ref. 55) without any justification; therefore, we investigated orders 2, 4, 6, 8, 10, 12, 14, 16, 18 and 20 for the filter. The Matlab function used to implement this step was *ellip*.

### Wavelet de-noising filter (Wavelet)

The wavelet transform is a powerful tool for signal and image processing that has been successfully applied to many scientific fields, such as signal processing, image compression, computer graphics, and pattern recognition^56,57^. Wavelet de-noising consists of three steps: wavelet decomposition, detail-coefficients thresholding, and reconstruction. In this study, the level *N* was set to 1, 2, 3, 4, 5, 6, 7, 8, 9, and 10. The Matlab function used to implement this step was *wden*.

### Code availability

The main Matlab function used to find the optimal filter is shown as follows.

function [filtered_PPG] = filter_configuration(raw_PPG,filter_type,order,Fs,fL,fH)

Fn = Fs/2;

switch filter_type

 

case 1

[A,B,C,D] = butter(order,[fL fH]/Fn);

[filter_SOS,g] = ss2sos(A,B,C,D);

filtered_PPG = filtfilt(filter_SOS,g,raw_PPG);

 

case 2

[A,B,C,D] = cheby1(order,0.1,[fL fH]/Fn);

[filter_SOS,g] = ss2sos(A,B,C,D);

filtered_PPG = filtfilt(filter_SOS,g,raw_PPG);

 

case 3

[A,B,C,D] = cheby2(order,20,[fL fH]/Fn);

[filter_SOS,g] = ss2sos(A,B,C,D);

filtered_PPG = filtfilt(filter_SOS,g,raw_PPG);

 

case 4

[A,B,C,D] = ellip(order,0.1,30,[fL fH]/Fn);

[filter_SOS,g] = ss2sos(A,B,C,D);

filtered_PPG = filtfilt(filter_SOS,g,raw_PPG);

 

case 5

d = fir1(order,[fL fH]/Fn,'bandpass');

filtered_PPG = filtfilt(d,1,raw_PPG);

 

case 6

d = designfilt('bandpassfir','FilterOrder',order,'StopbandFrequency1',fL-0.2,'PassbandFrequency1',fL,...

'PassbandFrequency2',fH,'StopbandFrequency2',fH+2,'DesignMethod','ls','SampleRate',sample_freq);

filtered_PPG = filtfilt(d,raw_PPG);

 

case 7

filtered_PPG = smooth(raw_PPG,order);

 

case 8

filtered_PPG = medfilt1(raw_PPG,order);

 

case 9

filtered_PPG= wden(raw_PPG,'modwtsqtwolog','s','mln',order,'db2');

 

end

end

## Additional information

**How to cite this article:** Liang, Y. *et al.* An optimal filter for short photoplethysmogram signals. *Sci. Data* 5:180076 doi: 10.1038/sdata.2018.76 (2018).

**Publisher’s note:** Springer Nature remains neutral with regard to jurisdictional claims in published maps and institutional affiliations.

## Figures and Tables

**Table 1:** The subjects’ demographic data.

Physical Index	Statistical Data
Females	115 (52%)
Age (years)	57±15
Height (cm)	161±8
Weight (kg)	60±11
Body Mass Index (kg/m^2^)	23±4
Systolic Blood Pressure (mm Hg)	127±20
Diastolic Blood Pressure (mm Hg)	71±11
Heart Rate (beats/min)	73±10
Hypertension	57 (26%)
Diabetes	38 (17.3%)
Vascular Infarction	29 (13.2%)
Note: Results are reported as the mean±standard deviation for quantitative variables and frequency distribution (%) for categorical variables. Disease information is provided per the subjects’ medical records.	
